# The Association Between Training Load and Injury Risk in Elite Youth Soccer Players: a Systematic Review and Best Evidence Synthesis

**DOI:** 10.1186/s40798-020-00296-1

**Published:** 2021-01-11

**Authors:** Sven Verstappen, Rogier M. van Rijn, Rick Cost, Janine H. Stubbe

**Affiliations:** 1 Codarts, University of the Arts, Kruisplein 26, 3012 CC Rotterdam, The Netherlands; 2 PErforming artist and Athlete Research Lab (PEARL), Rotterdam, The Netherlands; 3 Feyenoord Academy, Rotterdam, The Netherlands; 4 Feyenoord, Rotterdam, The Netherlands; 5 Erasmus MC University Medical Center, Department of General Practice, Rotterdam, The Netherlands; 6 Rotterdam Arts and Science Lab (RASL), Rotterdam, The Netherlands

**Keywords:** Workload, Injury, Football, Adolescent, Youth, Risk factor

## Abstract

**Background:**

Injury risk in elite youth soccer players is high. Implementing an optimal training load is of utmost importance to reduce the risk of injuries.

**Objective:**

To conduct a systematic review and best evidence synthesis to explore the effects of internal and external training load on injury risk in elite youth soccer players.

**Methods:**

MEDLINE, Embase, Web of Science, CENTRAL, and CINAHL were searched up until 17 January 2020. Each article had to meet all of the following criteria: (1) the study population consisted of male elite youth soccer players aged between 12 and 21 years; (2) a longitudinal, prospective study design was used; (3) soccer-related injuries were registered (i.e., self-reported or by medical staff); (4) external and/or internal load parameters were described; and (5) the article was published in an English peer-reviewed scientific journal. The quality of the included articles was assessed using the Newcastle–Ottawa Quality Assessment Scale (NOS). A best evidence synthesis was performed to rank the level of evidence.

**Results:**

Five studies (2 high quality, 3 low quality) were included. Best evidence synthesis highlighted that there was moderate evidence for (1) no association between 2-, 3-, and 4-week cumulative loads for total distance covered; (2) no association between 1-week workloads (sRPE × duration); and (3) no association between A:C workload ratios (4 weeks) and injury risk. For all other comparisons, only insufficient or conflicting evidence was found.

**Conclusion:**

There is a paucity of evidence for an association between internal and external training load parameters and injury risk in elite youth soccer players.

**Supplementary Information:**

The online version contains supplementary material available at 10.1186/s40798-020-00296-1.

## Key Points

- There is a lack of evidence for an association between internal and external training load parameters and injury risk in elite youth soccer players.
- Future high-quality longitudinal research is required to further assess the role of internal and external training load on the occurrence of injuries in elite youth soccer players, where a multifactorial approach must also be applied.

## Introduction

Soccer has evolved and games are played faster and more aggressively than in the past, requiring elevated fitness levels [1]. To meet these physical demands, soccer players have to be exposed to systematic and appropriate training regimes taking into account the balance between training load (TL; the combination of training volume, intensity, and frequency) and recovery [2]. TLs that are too low are insufficient to induce a functional, adaptive response. However, training too hard increases the risk of health problems, such as overtraining and injuries [3]. The high injury incidence in professional soccer players, ranging from 2.48 injuries [4] to 9.4 injuries [5] per 1000 h of exposure, suggests that finding the right balance between training, matches, and recovery is one of the biggest challenges soccer coaches and their staff face.

Adolescence, the period of life between childhood and adulthood, is a critical time in a soccer player’s career [6], and during this period, injuries can have a detrimental effect on future performance [7, 8] and career opportunities [9–12]. Unfortunately, injury risk in elite youth soccer players is even higher compared to their professional counterparts, ranging from 2.0 [13] to 19.4 injuries [14] per 1000 h of exposure, with match injury incidence as high as 48.7 injuries [14] per 1000 h of match exposure.

Implementing an optimal training load in elite youth soccer training programs is of utmost importance to reduce the risk of injuries and attain peak performance. Training load can be categorized into two components. External load refers to the physical work performed during training or matches, such as distance covered and accelerations [15], whereas internal load comprises the psychophysiological response to the external load [15]. Several studies in elite male soccer players have investigated the association between load and injury risk and concluded that players have an increased injury risk if the training load exceeds what their bodies can tolerate [16–20]. This association has received less attention in elite youth soccer players. While training may provoke positive training adaptations in youth athletes, it is also likely that they will respond differently from their adult counterparts to a particular training load, resulting in different fatigue, stress, injury, or illness responses [6]. Gabbett et al. determined the existing knowledge on the relationship between workload and injuries in adolescent male football players (i.e., American football, Australian rules football, soccer, rugby league, rugby union) and concluded that the balance between what is required to maintain or improve skill versus physiological performance and the association with injury risk is not well understood [6]. Therefore, the aim of our study was to conduct a systematic review exploring the effects of external and internal training load on injury risk in elite youth soccer players. This enables coaches and staff to better understand the impact of training load on injury risk in this specific target group.

## Methods

### Search Strategy

The systematic review was conducted according to the Preferred Reporting Items for Systematic Reviews and Meta-Analyses (PRISMA) guidelines [21]. Systematic literature searches were conducted in MEDLINE, Embase, Web of Science, the Cochrane Central Register of Controlled Trials (CENTRAL), and CINAHL (from the inception of databases up to 17 January 2020). The full search strategy is presented in Supplementary File 1.

Based on the title and abstract, two reviewers (SV, RMvR) selected the articles for full-text appraisal. From this pool, the two reviewers (SV, RMvR) independently selected articles for final inclusion. Each article had to meet all of the following criteria: (1) the study population consisted of male elite youth soccer players aged between 12 and 21 years, (2) a longitudinal prospective study design was used, (3) soccer-related injuries were registered (i.e., self-reported or by medical staff), (4) external and/or internal load parameters were described, and (5) the article was published in an English peer-reviewed scientific journal. Elite youth soccer players were defined as players who are part of an academy of an elite soccer club playing in the highest competition of their country. EndNote X8 software (Clarivate Analytics, Philadelphia, PA, USA) was used to perform the selection process. Any disagreement over inclusion was resolved via discussion between the reviewers. In the case of disagreement, a third reviewer (JHS) was consulted. The references of all included studies were checked for other relevant articles.

### Data Extraction

The following relevant data from each study were extracted: study details (author, year of publication, country, duration of follow-up), study population (sample size, age), injury definition, workload (external and internal load parameters), and measure of association (i.e., relative risk [RR] or odds ratio [OR]). Where possible, these associations were directly extracted from the original article. For articles in which this information was not presented, associations calculated using raw data were provided. Load parameters were classified as external or internal based on the International Olympic Committee consensus statement on load in sport and risk of injury [22]. The first reviewer (SV) extracted data from the included studies. In case of uncertainty, a second reviewer (RMvR) was consulted.

### Quality Assessment

The methodological quality of each study was assessed using an adapted version of the Newcastle–Ottawa Quality Assessment Scale (NOS) for cohort studies [23]. The original NOS consists of eight items and judges the quality of case–control and cohort studies. For cohort studies, the eight items can be grouped into three perspectives: selection of cohorts (4 items), comparability of cohorts (1 item), and assessment of outcome (3 items). A star-based rating system is used to indicate the quality of a study. A maximum of one star can be given for each item within the “Selection” and “Outcome” categories, and a maximum of two stars for the “Comparability” category, resulting in a maximum score of nine stars for high-quality studies. The original NOS was modified for the purpose of our review. In our modified version, two items of the original eight were deleted. Items 2 (selection of a non-exposed cohort) and 5 (assesses the comparability of cohorts) were deleted as our review focuses solely on male elite youth soccer players. In addition, we included a new item to the original scale, regarding injury definition, which was adopted from a modified version of the NOS used in a systematic review and meta-analysis of epidemiological data on injuries in professional male soccer [24]. Table 1 describes the seven criteria of our adapted version of the Newcastle–Ottawa Scale (NOS) for cohort studies. A study could be awarded a maximum of one star for each item if appropriate methods had been clearly reported, resulting in a maximum score of seven stars. The higher the number of stars given to a study, the lower the risk of bias. NOS scores were divided into high quality/low risk of bias (5–7 stars) and low quality/high risk of bias (0–4 stars).

**Table 1:** Description of the 7 criteria designed to assess the risk of bias of external validity quality in the studies

Criterion	Description of criteria
1. Definition of soccer-related injury	Studies that aimed to investigate soccer-related injuries should present a definition of an injury informing what was considered as an injury in the study. Studies that present a definition of time-loss injury received a star for this criterion.
2. Representativeness of the exposed cohort	(a) Truly representative of the average soccer players in the community^a^; (b) somewhat representative of the average soccer players in the community^a^; (c) selected group of users; (d) no description of the derivation of the cohort.
3. Ascertainment of exposure	(a) Secure record^a^; (b) structured interview^a^; (c) written self-report; (d) no description.
4. Demonstration that outcome of interest was not present at the start of the study	(a) Yes^a^; (b) no Studies that described that all soccer players included were injury-free at baseline received a star for this criterion.
5. Assessment of outcome	(a) Independent blind assessment^a^; (b) record linkage^a^; (c) self-report; (d) no description.
6. Was follow-up long enough for outcomes to occur?	(a) Yes^a^; (b) no Studies that carried out a follow-up period of at least 12 weeks received a star for this criterion.
7. Adequacy of follow-up of cohorts	(a) Complete follow-up of all subjects accounted for^a^; (b) subjects lost to follow-up unlikely to introduce bias (up to 20% loss) or description provided of those lost^a^; (c) follow-up rate < 80% and no description of those lost; (d) no statement.

^a^Articles with this alternative received a star for this criterion

Each included study was appraised by two authors (SV and RMvR) independently, and all discrepancies in scoring were resolved by arbitration between the two reviewers. In cases where discrepancies could not be resolved, a third reviewer (JHS) assessed the item in question. The reviewed studies were not blinded for reasons of practicality.

A best evidence synthesis was conducted to rate the strength of the evidence. The following ranking of evidence was used [25]:

1. Strong evidence: consistent findings in multiple (≥ 2) high-quality studies;
2. Moderate evidence: consistent findings in one high-quality study and at least one low-quality study, or consistent findings in multiple low-quality studies;
3. Insufficient evidence: only one study available; and
4. Conflicting evidence: inconsistent findings in multiple (≥ 2) studies.

Results of the studies reporting on a particular relationship were considered consistent when, for at least 75% of the study, results were in the same direction, as defined by *p* < 0.05.

## Results

### Search Results

**Fig. 1:**
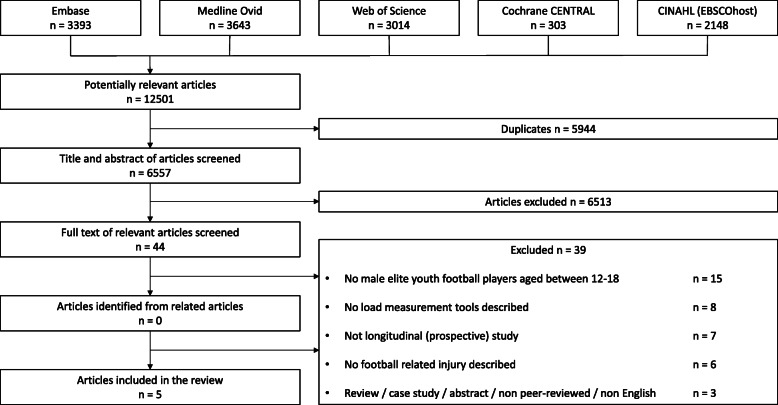
Flowchart of the selection process for inclusion of articles in the systematic review

An initial 12,501 articles were retrieved from database searches (Embase = 3393; MEDLINE Ovid = 3643; Web of Science = 3014; Cochrane CENTRAL = 303; CINAHL = 2148). After removal of 5944 duplicate records, a further 6557 irrelevant articles were removed based on title and abstract, resulting in 44 articles for full-text appraisal. Finally, five articles were included in the review (see Fig. 1).

### Description of the Included Studies

The five included studies accounted for a total participant pool of 270 elite male youth soccer players [26–30]. Follow-up periods ranged from one [30] to five [29] consecutive seasons. The sample size ranged from 22 to 122 soccer players. Two studies included external load measurements [26, 27], and three studies examined both external and internal load parameters [28–30]. The following load parameters were taken into account in the included studies: (1) internal load: psychosocial stress and recovery; (2) external load: total distance covered, high speed running, acceleration, duration, monotony, acute to chronic (AC) ratio (based on GPS and accelerometer-derived variables); and (3) combination of internal and external load parameters: workload, strain, and AC ratio (based on sRPE and duration). In four (out of five) studies, an injury definition was described. Definitions provided were (1) any physical complaint sustained by a player that results from a soccer match or training, resulting in time loss (unable to take full part in future soccer activities) or medical attention (> 1 day but still able to take full part in future soccer activities) [28], and (2) injury that occurred during a scheduled training session or match that caused absence from the next training session or match [27, 29, 30]. In three studies, injuries were registered by the medical staff [26–28], in one study by self-report [30], and in one study no description was provided [29]. A detailed description of the study characteristics is presented in Table 2.

**Table 2:** Characteristics of included studies

Study	Follow-up	Population	Injury definition	Workload	
				Internal	External
Bacon and Mauger [26] 2017 UK	2 seasons	*N* = 41 Mean age 17.8 ± 1.1 yrs	Overuse injuries—no definition described	n.a.	Total distance (m); weekly and whole season training/match averages High speed running (m); weekly and whole season training/match averages
Bowen et al. [27] 2017 UK	2 seasons	*N* = 32 Mean age 17.3 ± 0.9 yrs	A non-contact/contact injury was defined as one that caused any absence from future football participation	n.a.	Total distance (m); this includes walking, jogging, fast running, and sprinting High speed distance (m); total distance > 20 km/h Total load (au); acceleration along *x*, *y*, and *z* axes Accelerations (*n*); a change in GPS speed data for at least half a second with maximum acceleration in the period of at least 0.5 m/s/s
Brink et al. [28] 2010 Netherlands	*2* seasons	*N* = 53 Mean age 15–18 yrs	Any physical complaint sustained by a player that results from a soccer match or training, resulting in time loss (unable to take full part in future soccer activities) or medical attention (> 1 day but still able to take full part in future soccer activities) Traumatic injuries resulted from a specific, identifiable event. Overuse injuries resulted from repeated microtrauma without a single identifiable event	Rate of perceived exertion; global intensity of each training session rated on Borg 15-point scale Psychosocial stress and recovery; Recovery Stress Questionnaire for athletes (RESTQ-Sport)	Physical stress (min); duration of training sessions and matches Training load; product of the session-RPE and the physical stress Monotony; daily mean training load divided by the SD of the daily mean training load over a 1-week period Strain; product of weekly training load and monotony
Delecroix et al. [29] 2019 France	4 (U19) and 5 (U21) seasons	U19: *n* = 52; mean age 16.8 ± 0.9 yrs U21: *n* = 70; mean age 20.1 ± 0.3 yrs	Any physical complaint sustained by a player that resulted from a football match or training, that made the player unable to participate in a future football training or match	Rate of perceived exertion; global intensity of each training session and match rated on a 0–10-point scale	Workload (au); product of the session-RPE and duration of training session and matches A:C ratio (au); 1-week workload divided by the average workload of the last 28/21/14 days Week-to-week load changes; 1-week load divided by accumulated load of previous 7 days
Raya- González et al. [30] 2019 Spain	1 season	*N* = 22 Mean age 18.6 ± 0.6 yrs	A non-contact injury that occurred during a scheduled training session or match that caused absence from the next training session or match	Rate of perceived exertion; global intensity of each training session and match rated on a 0–10-point scale	Workload (au); the sum of load (RPE*duration) for all training sessions and matches for each week A:C ratio; sum of the current week’s load (acute load) divided by the average weekly training load over the previous four weeks (chronic load)

*yrs* years, *n.a.* not applicable, *m* meters, *km/h* kilometers per hour, *GPS* Global Positioning System, *au* arbitrary unit, *n* number, *min* minutes, *RESTQ* Recovery Stress questionnaire, *SD* standard deviation, *RPE* Rate of Perceived Exertion, *A:C ratio* acute to chronic workload ratio

### Risk of Bias Assessment and Best Evidence Synthesis

Of the five included studies, two were of high quality (low risk of bias) and three were of low quality (high risk of bias) (Table 3). Reviewers (SV, RMvR) retained agreement on all scoring and bias assessment results. For full details of the best evidence synthesis, see Tables 4, 5, and 6.

**Table 3:** Quality of included studies as assessed on the Newcastle–Ottawa Scale (NOS)

Study	NOS score								
	Selection				Outcome			Total	Risk of bias
	1	2	3	4	5	6	7		
Bacon and Mauger [26]	0	1	1	0	1	1	0	4	High
Bowen et al. [27]	1	1	1	0	1	1	0	5	Low
Brink et al. [28]	1	1	1	0	1	1	0	5	Low
Delecroix et al. [29]	1	1	1	0	0	1	0	4	High
Raya-Gonzales et al. [30]	1	1	1	0	0	1	0	4	High

**Table 4:** Results of the best evidence synthesis concerning contact and traumatic injuries

Risk factor	Study	Specification of independent variable	Outcome OR/RR (95% CI)	Best evidence synthesis	
				Presence of association	Level of evidence
Internal load parameters					
Psychosocial stress and recovery	Brink et al. [28]	General stress Emotional stress Social stress Conflicts/pressure Fatigue Lack of energy Physical complaints Success Social recovery Physical recovery General well-being Sleep quality Disturbed breaks Emotional exhaustion Fitness/injury Being in shape Personal accomplishment Self-efficacy Self-regulation	0.97 (0.74–1.27) 1.04 (0.76–1.44) 0.96 (0.70–1.30) 0.95 (0.74–1.22) 0.93 (0.72–1.20) 0.94 (0.70–1.26) 0.99 (0.74–1.33) 1.04 (0.81–1.35) 0.99 (0.78–1.26) 0.88 (0.69–1.13) 1.10 (0.85–1.42) 0.99 (0.77–1.27) 0.94 (0.72–1.22) 0.85 (0.67–1.09) **1.29 (1.01**–**1.66)** 0.91 (0.73–1.13) 1.06 (0.84–1.35) 1.00 (0.80–1.25) 0.90 (0.75–1.08)	No No No No No No No No No No No No No No Yes No No No No	Insufficient
External load parameters					
Total distance	Bowen et al. [27]	**Accumulated 1/2/3/4-week load**			Insufficient
		TD—low TD—moderately low TD—moderately high TD—high TD—very high	0.83/0.76/0.84/1.04 0.68/0.65/0.87/0.92 0.98/1.62/0.84/0.88 1.79/1.00/1.35/1.49 –/–/–/–	No/no/no/no No/no/no/no No/no/no/no No/no/no/no n.a	
High speed running	Bowen et al. [27]	**Accumulated 1/2/3/4-week load**			Insufficient
		HSD—low HSD—moderately low HSD—moderately high HSD—high HSD—very high	0.79/0.91/0.83/1.14 0.41/0.67/0.78/0.97 1.74/1.70/1.24/0.68 1.08/0.86/1.13/1.74 1.97/–/1.62/–	No/no/no/no No/no/no/no No/no/no/no No/no/no/no No/n.a./no/n.a.	
Acceleration	Bowen et al. [27]	**Accumulated 1/2/3/4-week load**			Insufficient
		ACC—low ACC—moderately low ACC—moderately high ACC—high ACC—very high	0.72/0.75/0.84/1.02 0.65/0.67/1.24/0.91 1.39/1.51/0.76/0.92 1.27/1.06/1.49/1.35 –/–/1.02/–	No/no/no/no No/no/no/no No/no/no/no No/no/no/no n.a./n.a./no/n.a.	
Duration	Brink et al. [28]	Duration (min) of training sessions and matches	**1.14 (1.06**–**1.23)**	Yes	Insufficient
Total load	Bowen et al. [27]	**Accumulated 1/2/3/4-week load**			Insufficient
		TL—low TL—moderately low TL—moderately high TL—high TL—very high	0.77/0.81/0.87/1.06 0.63/0.70/1.34/0.98 1.12/1.76/0.77/0.89 1.42/0.33/0.95/1.43 **4.84**/3.04/2.68/–	No/no/no/no No/no/no/no No/no/no/no No/no/no/no Yes/no/no/no	
Acute to chronic workload ratio (ACWR)	Bowen et al. [27]	**Overall/low chronic/high chronic**			Insufficient
		TD—low TD—moderately low TD—moderately high TD—high TD—very high HSD—low HSD—moderately low HSD—moderately high HSD—high HSD—very high ACC—low ACC—moderately low ACC—moderately high ACC—high ACC—very high TL—low TL—moderately low TL—moderately high TL—high TL—very high	0.37/0.26/0.62 1.72/2.12/1.47 0.44/0.44/0.91 1.22/2.80/0.91 **4.98**/–/3.79 0.32/0.24/1.91 1.45/0.82/1.52 1.32/2.55/0.69 0.49/0.85/0.54 2.28/–/3.62 0.39/0.25/– 1.75/1.79/1.66 0.79/0.59/1.03 1.47/2.48/0.64 **4.98**/–/5.91 0.38/0.21/0.60 **1.92**/1.64/1.97 0.87/0.77/0.86 1.20/2.280.32 2.74/–/6.12	No/no/no No/no/no No/no/no No/no/no Yes/n.a./no No/no/no No/no/no No/no/no No/no/no No/no/no No/no/n.a. No/no/no No/no/no No/no/no Yes/n.a./no No/no/no yes/no/no No/no/no No/no/no No/n.a./no	
Combined internal/external load parameters					
Workload	Brink et al. [28]	Product of the session-RPE and duration	**1.01 (1.00**–**1.02)**	Yes	Insufficient
Monotony	Brink et al. [28]	Daily mean training load divided by the SD of the daily mean training load over a 1-week period	**2.59 (1.22**–**5.50)**	Yes	Insufficient
Strain	Brink et al. [28]	Product of weekly training load and monotony	**1.01 (1.00**–**1.01)**	Yes	Insufficient

In bold are statistically significant (*p* < 0.05) associations

*OR* odds ratio, *RR* risk ratio, *CI* confidence interval, *TD* total distance, *HSD* high speed distance, *ACC* acceleration, *TL* total load, *min* minutes, *ACWR* acute to chronic workload ratio, *n.a.* not applicable, *RPE* rate of perceived exertion, *SD* standard deviation

**Table 5:** Results of the best evidence synthesis concerning non-contact and overuse injuries

Risk factor	Study	Specification of independent variable	Outcome OR/RR (95% CI)	Best evidence synthesis	
				Presence of association	Level of evidence
Internal load parameters					
Psychosocial stress and recovery	Brink et al. [28]	General stress Emotional stress Social stress Conflicts/pressure Fatigue Lack of energy Physical complaints Success Social recovery Physical recovery General well-being Sleep quality Disturbed breaks Emotional exhaustion Fitness/injury Being in shape Personal accomplishment Self-efficacy Self-regulation	1.03 (0.75–1.43) 1.24 (0.85–1.82) 1.07 (0.74–1.53) 0.94 (0.70–1.27) 0.96 (0.70–1.30) 1.07 (0.75–1.52) 1.02 (0.71–1.45) 0.76 (0.55–1.04) 0.94 (0.71–1.26) 0.89 (0.66–1.19) 0.93 (0.68–1.26) 0.86 (0.64–1.16) 1.00 (0.73–1.37) 0.92 (0.69–1.23) **1.46 (1.09**–**1.96)** 0.84 (0.64–1.09) 0.90 (0.68–1.20) 0.97 (0.74–1.27) 0.82 (0.66–1.02)	No No No No No No No No No No No No No No Yes No No No No	Insufficient
External load parameters					
Total distance	Bacon and Mauger [26]	**2-week cumulative loads**			Insufficient Moderate (2-, 3-, 4-week cumulative loads)
		TD—normal load TD—low load TD—high load **3-week cumulative loads** TD—normal load TD—low load TD—high load **4-week cumulative loads** TD—normal load TD—low load TD—high load	(ref.) 1.26 (0.16–9.77) 0.67 (0.40–1.14) (ref.) 0.69 (0.29–1.64) 0.95 (0.44–2.05) (ref.) 0.69 (0.29–1.64) 0.95 (0.44–2.05)	No No No No No No	
	Bacon and Mauger [26]	TD (low, normal, high)	**0.0029** (0.0029–0.003)	Yes	
	Bowen et al. [27]	**Accumulated 1/2/3/4-week loads**			
		TD—low TD—moderate-low TD—moderate-high TD—high TD—very high	**0.30**/0.61/0.67/1.01 1.45/0.95/0.87/0.77 0.83/1.19/1.08/1.06 1.64/1.37/1.65/1.55 3.04/3.35/2.79/2.30	Yes/no/no/no No/no/no/no No/no/no/no No/no/no/no No/no/no/no	
High speed running	Bacon and Mauger [26]	**2-week cumulative loads**	.		Insufficient Conflicting (2-, 4-week cumulative HSD loads) Moderate(3-week HSD loads)
		HSR—normal load HSR—low load HSR—high load **3-week cumulative loads** HSR—normal load HSR—low load HSR—high load **4-week cumulative loads** HSR—normal load HSR—low load HSR—high load	(ref.) 0.99 (0.38–2.59) 0.58 (0.33–1.02) (ref.) 0.51 (0.21–1.21) 1.05 (0.54–2.03) (ref.) 0.51 (0.21–1.21) 1.05 (0.54–2.03)	No No No No No No	
	Bacon and Mauger [26]	HSR (low, normal, high)	**0.065 (**0.064–0.067)	Yes	
	Bowen et al. [27]	**Accumulated 1/2/3/4-week loads**			
		HSD—low HSD—moderately low HSD—moderately high HSD—high HSD—very high	0.54/**0.26**/0.68/0.94 1.10/0.95/0.79/0.61 **1.73**/1.42/1.40/**2.14** 0.65/1.75/1.42/0.68 0.00/0.00/0.00/0.59	No/yes/no/no No/no/no/no Yes/no/no/yes No/no/no/no No/no/no/no	
Acceleration	Bowen et al. [27]	**Accumulated 1/2/3/4-week loads**			Insufficient
		ACC—low ACC—moderately low ACC—moderately high ACC—high ACC—very high	0.47/0.60/0.69/0.95 0.77/0.96/0.68/0.72 1.03/1.27/1.29/1.02 **2.25**/1.10/1.47/1.64 1.31/**4.25**/**5.11**/**4.25**	No/no/no/no No/no/no/no No/no/no/no Yes/no/no/no No/yes/yes/yes	
Duration	Brink et al. [28]	Duration (min) of training sessions and matches	1.07 (0.98–1.18)	No	Insufficient
Total load	Bowen et al. [27]	**Accumulated 1/2/3/4-week loads**			Insufficient
		TL—low TL—moderately low TL—moderately high TL—high TL—very high	**0.31**/0.59/0.55/0.80 1.40/1.17/0.85/1.04 0.79/1.13/1.37/0.94 **2.20**/1.45/1.41/1.64 0.00/0.00/1.39/1.07	yes/no/no/no No/no/no/no No/no/no/no Yes/no/no/no No/no/no/no	
ACWR	Bowen et al. [27]	**Overall/low chronic/high chronic**			Insufficient
		TD—low TD—moderately low TD—moderately high TD—high TD—very high HSD—low HSD—moderately low HSD—moderately high HSD—high HSD—very high ACC—low ACC—moderately low ACC—moderately high ACC—high ACC—very high TL—low TL—moderately low TL—moderately high TL—high TL—very high	1.50/0.29/1.19 0.96/1.12/0.62 1.45/1.43/1.53 1.05/1.28/1.51 0.00/–/– 0.60/0.63/1.20 0.88/0.88/0.81 1.33/0.85/**2.09** 1.39/**2.55**/0.47 –/–/– 1.22/0.31/1.37 0.81/1.32/0.63 1.52/1.23/1.49 1.41/1.30/1.54 –/–/– 1.20/0.50/0.98 0.84/0.84/0.79 **1.87**/1.55/1.93 0.87/1.16/0.53 –/–/–	No/no/no No/no/no No/no/no No/no/no No/n.a./n.a. No/no/no No/no/no No/no/yes No/yes/no n.a./n.a./n.a No/no/no No/no/no No/no/no No/no/no n.a./n.a./n.a No/no/no No/no/no Yes/no/no No/no/no n.a./n.a./n.a	
Combined internal/external load parameters					
Workload	Brink et al. [28]	Product of the session-RPE and duration	1.01 (1.00–1.02)	No	Insufficient
Monotony	Brink et al. [28]	Daily mean training load divided by the SD of the daily mean training load over a 1-week period	0.84 (0.25–2.76)	No	Insufficient
Strain	Brink et al. [28]	Product of weekly training load and monotony	1.00 (1.00–1.01)	No	Insufficient

In bold are statistically significant (*p* < 0.05) associations

*OR* odds ratio, *RR* risk ratio, *CI* confidence interval, *ref* reference, *TD* total distance, *HSD* high speed distance, *ACC* acceleration, *TL* total load, *HSR* high speed running, *min* minutes, *ACWR* Acute Chronic Workload Ratio, *n.a.* not applicable, *RPE* rate of perceived exertion, *SD* standard deviation

**Table 6:** Results of the best evidence synthesis concerning all injuries

Risk factor	Study	Specification of independent variable	Outcome OR/RR (95% CI)	Best evidence synthesis	
				Presence of association	Level of evidence
External load parameters					
Total distance	Bowen et al. [27]	**Accumulated 1/2/3/4-week loads**			Insufficient
		TD—low TD—moderately low TD—moderately high TD—high TD—very high	**0.25**/0.62/0.53/0.89 1.38/0.76/1.23/0.73 0.95/**1.55**/1.36/1.19 1.57/1.27/1.31/**1.64** 2.59/2.88/2.37/1.29	Yes/no/no/no No/no/no/no No/yes/no/no No/no/no/yes No/no/no/no	
High speed running	Bowen et al. [27]	**Accumulated 1/2/3/4-week loads**			Insufficient
		HSD—low HSD—moderately low HSD—moderately high HSD—high HSD—very high	**0.38**/**0.30**/0.67/0.79 1.16/0.81/0.84/0.73 **1.73**/**1.72**/1.15/**1.56** 0.59/1.45/**1.66**/1.26 0.82/0.00/0.33/0.33	Yes/yes/no/no No/no/no/no Yes/yes/no/yes No/no/yes/no No/no/no/no	
Acceleration	Bowen et al. [27]	**Accumulated 1/2/3/4-week loads**			Insufficient
		ACC—low ACC—moderately low ACC—moderately high ACC—high ACC—very high	**0.35**/0.51/0.63/0.93 1.01/0.92/0.77/0.82 1.00/1.21/1.32/1.01 **1.83**/1.37/1.38/**1.66** **3.06**/**3.19**/**3.84**/2.37	Yes/no/no/no No/no/no/no No/no/no/no Yes/no/no/yes Yes/yes/yes/no	
Total load	Bowen et al. [27]	**Accumulated 1/2/3/4-week loads**			Insufficient
		TL—low TL—moderately low TL—moderately high TL—high TL—very high	**0.27**/0.50/0.55/0.75 1.45/1.07/0.98/1.01 0.98/1.38/1.39/1.12 **1.65**/1.03/1.09/1.20 2.00/1.93/1.59/1.84	Yes/no/no/no No/no/no/no No/no/no/no Yes/no/no/no No/no/no/no	
ACWR	Bowen et al. [27]	**Overall/low chronic/high chronic**			Insufficient
		TD—low TD—moderately low TD—moderately high TD—high TD—very high HSD—low HSD—moderately low HSD—moderately high HSD—high HSD—very high ACC—low ACC—moderately low ACC—moderately high ACC—high ACC—very high TL—low TL—moderately low TL—moderately high TL—high TL—very high	1.00/**0.28**/0.91 1.25/1.43/0.98 0.97/0.97/1.19 1.13/1.76/1.21 2.09/–/1.80 **0.47**/0.47/1.52 1.10/0.86/1.11 1.32/1.30/1.27 0.98/1.82/0.50 0.95/–/1.63 0.85/**0.29**/0.71 1.16/1.49/1.04 1.15/0.94/1.25 1.44/1.70/1.10 2.09/–/2.71 0.84/0.37/0.81 1.15/1.15/1.22 1.34/1.16/1.34 1.01/1.59/0.43 1.17/–/2.67	No/yes/no No/no/no No/no/no No/no/no No/n.a./no Yes/no/no No/no/no No/no/no No/no/no No/n.a./no No/yes/no No/no/no No/no/no No/no/no No/n.a./no No/no/no No/no/no No/no/no No/no/no No/n.a./no	
Combined internal/external load parameters					
Workload	Delecroix et al. [29]	**U19**			Insufficient Moderate (1-week workload)
		1-week workload 2-week cumulative workload 3-week cumulative workload 4-week cumulative workload **U21** 1-week workload 2-week cumulative workload 3-week cumulative workload 4-week cumulative workload	1.11 (0.84–1.50) 1.03 (0.77–1.38) 0.97 (0.74–1.28) 1.00 (0.76–1.33) 1.18 (0.92–1.52) 1.28 (0.97–1.69) **1.39 (1.04**–**1.84)** **1.40 (1.06**–**1.86)**	No No No No No No Yes Yes	
	Raya-Gonzálaz et al. [30]	Sum of load (RPE*duration) for all training sessions and matches for each week	1.00 (0.99–1.00)^a^	No	
ACWR	Delecroix et al. [29]	**U19**			Insufficient Moderate (4-week A:C workload)
		2-week A:C workload 3-week A:C workload 4-week A:C workload Week-to-week workload changes **U21** 2-week A:C workload 3-week A:C workload 4-week A:C workload Week-to-week workload changes	0.99 (0.90–1.09) 1.01 (0.95–1.06) 1.01 (0.96–1.07) 1.00 (0.96–1.04) 0.86 (0.58–1.29) 0.88 (0.66–1.16) 0.89 (0.71–1.13) 1.00 (0.95–1.06)	No No No No No No No No	
	Raya-Gonzálaz et al. [30]	Sum of the current week’s load (acute load) divided by the average weekly training load over the previous four weeks (chronic load)	0.16 (0.01–1.84)^a^	No	

In bold are statistically significant (*p* < 0.05) associations

*OR* odds ratio, *RR* risk ratio, *CI c*onfidence interval, *ref* reference, *TD* total distance, *HSD* high speed distance, *ACC* acceleration, *TL* total load, *HSR* high speed running, *min* minutes, *ACWR* acute to chronic workload ratio, *n.a.* not applicable, *RPE* rate of perceived exertion, *SD* standard deviation, *U19* under 19 years squad, *U21* under 21 years squad, *A:C* acute to chronic

^a^90% confidence interval

### Evaluation of Internal Load Parameters

#### Psychosocial Stress and Recovery

Insufficient evidence exists for an association between psychosocial stress and recovery, and traumatic and overuse injury risk [28]. This high-quality study used the Recovery Stress Questionnaire for Athletes (RESTQ-Sport77) and concluded that the subscale fitness/injury was associated with the occurrence of traumatic and overuse injuries. Insufficient evidence also exists for a lack of an association between the sum score of the RESTQ-Sport, the subscale stress, and the subscale recovery and traumatic and overuse injury risks.

### Evaluation of External Load Parameters

#### Total Distance Covered

One high-quality study provided insufficient evidence for a lack of an association between total distance (TD) covered and injury risk (contact/traumatic) [27]. Two studies, one high quality and one low quality, investigated the association between TD covered and the occurrence of non-contact/overuse injuries [26, 27]. Both studies found no significant effect of 2-, 3-, and 4-week cumulative loads for TD on injury risk, resulting in moderate evidence for no association between these factors. Being in a higher TD loading group lowered the risk of an overuse injury, although insufficient evidence for this relationship exists [26]. Insufficient evidence also exists for a low, 1-weekly TD covered (0–8811 m) reducing the risk of non-contact/overuse injuries [27]. A low, 1-weekly TD (0–8811 m) reduced the risk of overall injury in one study only, therefore offering insufficient evidence. A moderately high, 2-weekly TD covered (39,806–58,405 m) and a high 4-weekly TD covered (112,244–143,917 m) increased the risk of overall injury, but this was also only found in one study resulting in insufficient evidence [27]. For all other comparisons, insufficient evidence was found for a lack of association.

#### High Speed Running

Insufficient evidence exists for a lack of a relationship between high speed running distance (HSD), accumulated over 1–4 weeks, and the occurrence of contact/traumatic injuries [27].

Two studies, one high quality and one low quality, investigated the association between HSD and non-contact/overuse injury risk [26, 27]. Being in a higher HSD loading group lowered the risk of an overuse injury, although this was only found in one study leading to insufficient evidence for this effect [26]. Two-, 3-, and 4-week cumulative loads of HSD were not significantly associated with an increased risk of overuse injury in the study of Bacon et al. [26]. A study by Bowen and colleagues showed that low 2-weekly (0–755 m) and moderately high 4-weekly HSD (3502–5122 m) was significantly related with non-contact/overuse injury occurrence [27], whereas no association was found for 3-weekly HSD loads [27]. Therefore, there is conflicting evidence for an association between 2- and 4-week accumulated HSD loads and non-contact/overuse injury risk, and moderate evidence for no association between 3-week accumulated HSD loads and injury occurrence. There is insufficient evidence for an association between moderately high 1-weekly HSD loads (856–1448 m) and non-contact/overuse injury risk. There is also insufficient evidence for an association between (1) low 1- and 2-weekly HSD loads; (2) moderately high 1-, 2-, and 4-weekly HSD loads; and (3) high 3-weekly HSD loads and the occurrence of overall injuries [27].

#### Acceleration

One high-quality study investigated the relationship between the number of accelerations (ACC) and injury occurrence [27]. Insufficient evidence was found for no association between ACC, accumulated over 1–4 weeks, and the occurrence of contact/traumatic injuries. Insufficient evidence was also found for an association between very high ACC (> 9254), accumulated over 2–4 weeks, and high weekly ACC (2558–3474) loads and the occurrence of non-contact/overuse injuries. Moreover, there is insufficient evidence for an association between (1) low weekly ACC loads, (2) high 1- and 4-weekly ACC loads, and (3) very high 1–3-weekly ACC loads and overall injury occurrence. For all other comparisons, insufficient evidence exists for a lack of an association.

#### Duration

One high-quality study evaluated the association between the duration of training and matches (over the preceding week, calculated in hours) and traumatic and overuse injury risk [28]. Weekly duration was significantly higher for players with a traumatic injury compared to healthy players, resulting in insufficient evidence for an association as only one study reported this. Insufficient evidence also exists for no correlation between duration and overuse injury risk.

#### Acute to Chronic Workload Ratio (ACWR)

One high-quality study investigated the association between A:C workload ratios (based on GPS and accelerometer-derived variables) and the occurrence of contact/traumatic, non-contact/overuse, and overall injuries [27]. The risk of contact injury was increased when the overall A:C total distance (TD) ratio was ≥ 1.76. For low chronic TDs covered (< 22 335 m), overall injury occurrence was associated with a low A:C TD ratio (0–0.32). In addition, there is insufficient evidence for (1) an association between very high A:C TD ratios and contact injury occurrence, (2) an association between a low A:C TD ratio and overall injury occurrence (low chronic TD covered), and (3) no association for all other comparisons.

For low chronic high speed running distance (HSD) (< 938 m), non-contact injury risk was increased by a high A:C ratio (1.41–1.96). For high chronic HSDs (> 938 m), non-contact injury occurrence was associated with a moderately high A:C ratio (0.91–1.34). However, a low ratio (0–0.36) for all chronic HSDs was associated with a reduced overall injury risk. Moreover, there is insufficient evidence for (1) an association between high A:C HSD ratios and non-contact injury occurrence (low chronic HSDs), (2) an association between a moderately high A:C HSD ratio and non-contact injury occurrence (high chronic HSDs), (3) an association between low A:C HSD ratios and overall injury occurrence, and (4) no association for all other comparisons.

The risk of contact injury was increased when the A:C acceleration (ACC) ratio was > 1.77 (very high). Overall injury risk was reduced when the A:C ACC ratio was low (0–0.33) for low chronic ACC (< 1856). Furthermore, there is insufficient evidence for (1) an association between very high A:C ACC ratios and contact injury occurrence, (2) an association between a low A:C ACC ratio and reduced overall injury occurrence (low chronic ACCs), and (3) no association for all other comparisons.

The risk of non-contact injury was increased when the A:C total load (TL) ratio was moderately high (0.88–1.32). A moderately low A:C TL ratio (0.44–0.88) was associated with the occurrence of contact injuries. In sum, there is insufficient evidence for (1) an association between moderately high A:C TL ratios and non-contact injury occurrence, (2) an association between moderately low A:C TL ratios and contact injury occurrence, and (3) no association for all other comparisons.

### Evaluation of Combined Internal and External Load Parameters

#### Workload

Three studies, one high quality and two low quality, investigated workload as a risk factor for injury occurrence [28–30]. All three studies defined workload as the product of the rate of perceived exertion (sRPE) during a session and the session’s duration. In the studies of Delecroix et al. and Raya-González et al., the sRPE was measured using a 10-point scale [29, 30], while Brink and colleagues used a 15-point Borg scale [28]. There was insufficient evidence for an association between workload and contact/traumatic injuries and also insufficient evidence for no association between workload and non-contact/overuse injuries [28].

There was moderate evidence for a lack of an association between 1-week workloads and overall injury risk [29, 30]. Delecroix and colleagues also calculated 2-, 3-, and 4-week cumulative workloads and found an association for 3- and 4-week cumulative workloads and injury occurrence in their under-21 (U21) squad, resulting in insufficient evidence for an association between 3- and 4-week cumulative workloads, and insufficient evidence for no association between 2- week cumulative workloads and overall injuries [29].

#### Monotony

One high-quality study described the association between the monotony of training load and the occurrence of traumatic injuries, as well as overuse injuries [28]. There is insufficient evidence for an association between monotony and traumatic injuries, and insufficient evidence for no association between monotony and overuse injuries.

#### Strain

There is insufficient evidence for an association between the amount of effort (strain) a player experienced and the occurrence of traumatic injuries, and also insufficient evidence for no association between strain and overuse injuries [28].

#### Acute to Chronic Workload Ratio (ACWR)

Two low-quality studies investigated the association between the acute to chronic (A:C) workload ratio and the occurrence of overall injuries [29, 30]. Delecroix et al. calculated A:C ratios (week-to-week, 2 weeks, 3 weeks, and 4 weeks) by dividing the 1-week workload (product of the sRPE and duration of training session and matches) by the average workload of the last 28/21/14 days [29]. In the under-19s (U19), as well as in the U21 squad, no associations were found. Raya-González and colleagues found no association between A:C workload (sRPE × duration) ratios (where acute refers to the current week and chronic refers to the rolling 4-week average) and injury occurrence [30]. This resulted in moderate evidence for no association between A:C workload ratios (4 weeks) and the occurrence of injuries and insufficient evidence for no association for A:C ratios (week-to-week, 2 weeks, and 3 weeks).

## Discussion

This systematic review evaluates the evidence concerning the effects of internal and external training load on injury risk in elite youth soccer players. According to the best evidence synthesis, there was insufficient evidence for an association between psychosocial stress and recovery (internal load) and the occurrence of injuries.

With regard to external load parameters, there was moderate evidence for no association between 2-, 3-, and 4-week cumulative loads for total distance covered and injury risk. Conflicting evidence exists for the association between 2- and 4-week accumulated HSD loads and non-contact/overuse injury risk. Moreover, there was moderate evidence for a lack of an association between 3-weekly accumulated HSD loads and injury occurrence. Furthermore, insufficient evidence existed for an association between the number of accelerations, duration of training/matches, A:C workload ratios (based on GPS and accelerometer-derived variables), and the occurrence of injuries.

Combined load parameters, such as monotony and strain, showed insufficient evidence for an association with injury risk. There was moderate evidence for no correlation between 1-week workloads (product of sRPE and the duration) and overall injury risk, alongside insufficient evidence for an association between 3- and 4-week cumulative workloads and injuries. Finally, moderate evidence existed for a lack of an association between A:C workload ratios (4 weeks) and the occurrence of injuries, with insufficient evidence also found for no association for A:C ratios (week-to-week, 2 weeks, and 3 weeks).

Overall, the evidence for associations between internal/external load factors and the occurrence of injuries is based on a limited number of studies (*n* = 5), with a maximum of three studies per potential risk factor, which limits the conclusions that can be drawn.

In addition, the risk of bias assessment resulted in two studies being assessed as having a low risk of bias and three studies being assessed as having a high risk of bias. Critical items in the risk of bias assessment were the items on absence of injuries at the start of the study (item 4) and adequacy of follow-up of cohorts (item 7). None of the studies demonstrated that included soccer players were injury-free at baseline. Moreover, none of the studies reported a follow-up rate or provided a description of those soccer players who were lost between inception and follow-up. These studies are therefore more susceptible to selection and attrition bias, and, as a consequence, this will affect the generalizability of our results.

In addition to the shortcomings mentioned above, there are some other explanations for the low evidence found in this systematic review. All included studies used a general injury definition, for example “Any physical complaint sustained by a player that results from a soccer match or training, resulting in time loss or medical attention” or “A non-contact/contact injury was defined as one that caused any absence from future soccer participation” [27, 28]. Using a general injury definition will lead to the inclusion of all types of injuries. For example, muscle injuries are the most common non-contact related (overuse) injuries in soccer and constitute approximately 30 to 60% of all time-loss injuries in elite youth soccer players [14, 31–33]. Selecting a more homogenous group of injured players based on a more specific injury definition (i.e., muscle injuries), taking into account the injury mechanism, will probably lead to a clearer view on the true association between workload and injury risk. Furthermore, there is a possibility that the association between training load and injury risk alone is not that strong and that other factors, the interplay between them, or the mediating effects of them are more important in a population of elite youth soccer players. This is underlined by Pfirrmann and colleagues who conducted a systematic review to compare the injury incidences and characteristics of male professional adult and elite youth soccer players [34]. They summarized that factors, such as age, playing position, season, concealment of injury, multiple injuries, imbalance between external pressure and internal effort, training, recovery time, re-injuries, and maturity status, can lead to higher injury incidences. The multifactorial nature of injuries needs to be addressed, which is confirmed by the model of Meeuwisse et al. that highlighted the importance of accounting for all factors involved, such as the internal and external risk factors as well as the inciting event [35, 36]. Future studies investigating the association between internal and external training load and injury risk should also take these factors into account, and use a multifactorial approach to unravel the association between several risk factors and injuries.

To our knowledge, this is the first systematic review summarizing the available evidence for associations between internal and external training load and injury occurrence in elite youth soccer players. A strength of this systematic review is that a comprehensive literature search was conducted in five different databases, which makes it likely that all potential relevant studies have been identified. Alongside this, a best evidence synthesis was applied to summarize and evaluate the existing evidence. This methodology results in transparency in the process of evidence assessment by applying clear criteria that include the quality of the studies. However, this study also has some limitations. First, the studies included in this systematic review only included elite youth soccer players from Europe (UK, Spain, France, and the Netherlands), resulting in more homogenous groups of soccer players and possibly reducing generalizability to other parts of the world. Therefore, we would like to emphasize that more research is needed regarding internal and external workload and injury risk in elite youth soccer players. Second, different operational definitions of injury are used in the included studies. This variability of definitions can impact injury estimates and, as a result, influence the association between training load and injury risk. Third, there is the potential for a publication and language bias given that only published literature, written in English, was included for the purposes of this review.

## Conclusion

After summarizing the literature, it can be concluded that there is a paucity of evidence for an association between internal and external training load parameters and injury risk in elite youth soccer players. Future high-quality longitudinal research is required to further assess the role of internal and external training load on the occurrence of injuries in elite youth soccer players, where a multifactorial approach must also be applied. In addition, the shortcomings of the included studies concerning selection and attrition bias should be taken into account.

## Supplementary Information

**Additional file 1.** Search strategy

## Data Availability

Not applicable.
