# Supplementary material for: The Association Between Training Load and Injury Risk in Elite Youth Soccer Players: a Systematic Review and Best Evidence Synthesis
Source: Sports Med Open. 2021 Jan 11;7:6. doi: 10.1186/s40798-020-00296-1 (PMC7801562; doi:10.1186/s40798-020-00296-1)
Supplement: Supplementary file 1 — Additional file 1. Search strategy [file 40798_2020_296_MOESM1_ESM.docx]

**Supplementary File 1. Search strategy**

| 1. Sport | ('soccer'/de OR 'soccer player'/de OR (soccer* OR (football* NOT (american-football* OR australian-football*))):ab,ti) |
| --- | --- |
| 2. Monitoring | ('monitoring'/de OR 'physiologic monitoring'/exp OR 'biological monitoring'/de OR 'stress'/exp OR 'workload'/de OR 'performance'/de OR 'fitness'/de OR 'metabolic capacity'/exp OR 'fatigue'/de OR 'exhaustion'/de OR 'muscle fatigue'/de OR 'dose response'/de OR 'sleep'/exp OR 'wellbeing'/exp OR 'mental health'/de OR 'psychological well-being'/de OR 'exercise recovery'/de OR 'quality of life'/exp OR 'injury'/de OR 'leg injury'/exp OR 'musculoskeletal injury'/exp OR 'rupture'/exp OR 'sport injury'/exp OR 'concussion'/exp OR 'burnout'/exp OR 'risk'/exp OR 'accident'/de OR 'pain'/exp OR 'pain assessment'/exp OR 'global positioning system'/exp OR 'geographic information system'/de OR 'heart rate measurement'/exp OR 'heart rate'/exp OR 'exercise intensity'/de OR (monitoring OR ((internal* OR external* OR training OR exercise OR game OR match OR competition OR physical* OR physiological*) NEAR/3 (load* OR demand*)) OR stress* OR workload OR work-load OR work-rate OR intensity OR (intense* NEAR/3 (training OR sport* OR exercise)) OR performance* OR fitness OR capacity OR ((metabolic OR aerobic OR anaerobic) NEAR/3 (power OR work OR load)) OR (activity* NEAR/3 profile*) OR ability* OR freshness OR fatigue OR exhaustion OR readiness OR preparedness OR (dose NEAR/3 response*) OR sleep* OR wellbeing OR well-being OR wellness OR ((mental OR psychology*) NEAR/3 (health OR state OR status)) OR recover* OR soreness OR readiness OR tiredness OR (quality NEAR/3 life) OR injury* OR rupture* OR concussion* OR overuse* OR trauma* OR illness OR overreach* OR overtrain* OR burnout OR burn-out OR risk* OR accident* OR incident* OR pain* OR ((total OR covered*) NEAR/3 distance*) OR acceleration* OR (high NEAR/3 speed NEAR/6 (running OR distance*)) OR (player NEAR/3 load) OR (maximal NEAR/3 velocity) OR Global-Positioning-System OR gps OR heart-rate* OR sprain* OR strain* OR (Borg NEAR/3 scale) OR (perceive* NEAR/3 exertion*) OR rpe):ab,ti) |
| 3. Population | ('elite athlete'/de OR 'professional development'/de OR 'professional competence'/de OR 'professionalism'/de OR (elite OR academy OR professional* OR semiprofessional* OR top-level OR talented):ab,ti) NOT ((female/exp OR (female* OR girl* OR woman OR women):ab,ti) NOT (male/exp OR (male OR boys OR men OR man):ab,ti)) NOT ([Conference Abstract]/lim OR [Letter]/lim OR [Note]/lim OR [Editorial]/lim) |
| 4. Limitations | [english]/lim + humans |
